# Predictive Value of the Red Cell Distribution Width‐To‐Albumin Ratio for Clinical Outcomes in Patients With Peptic Ulcer Perforation

**DOI:** 10.1002/wjs.12515

**Published:** 2025-02-24

**Authors:** Suleyman Utku Celik, Yasin Gulap, Mehmet Bahadir Demir, Mehmet Mert Demircioglu, Hilmi Erencan Polat, Sacit Altug Kesikli

**Affiliations:** ^1^ Department of General Surgery Gulhane Training and Research Hospital Ankara Turkey; ^2^ Department of General Surgery Ankara University School of Medicine Ankara Turkey

**Keywords:** albumin, mortality, peptic ulcer perforation, prognosis, red cell distribution width

## Abstract

**Background:**

Peptic ulcer perforation is a potentially life‐threatening complication of peptic ulcer disease. Several scoring systems have been developed to predict outcomes in these patients. The red cell distribution width‐to‐albumin ratio (RAR) has shown promise as a prognostic marker in various conditions, yet its role in peptic ulcer perforation remains unclear. This study aimed to evaluate the predictive value of RAR in patients with peptic ulcer perforation.

**Methods:**

This retrospective study was conducted between 2016 and 2024 on patients who underwent surgery for peptic ulcer perforation. Patient demographics, clinical features, laboratory values, and surgical outcomes were analyzed. The main outcomes were major postoperative complications and 30‐day mortality. Multivariate regression analysis was used to identify independent predictors of these outcomes. The ability of RAR to predict outcomes was also assessed.

**Results:**

The study included 187 patients with a median age of 49.7 years, of whom 78.6% were males. Major complications occurred in 18.1% of the patients and the 30‐day mortality rate was 9.6%. Multivariate analysis identified age, surgical delay, elevated C‐reactive protein and RAR as independent predictors of major complications. For 30‐day mortality, only age and RAR remained significant in the multivariate model. Receiver operating characteristic curve analysis showed that RAR had high diagnostic accuracy for predicting both major complications (AUC = 0.883) and mortality (AUC = 0.944).

**Conclusion:**

With its high sensitivity and specificity for predicting major complications and mortality in patients with peptic ulcer perforation, RAR has significant potential as a prognostic marker in conjunction with traditional risk factors in clinical practice.

## Graphical abstract

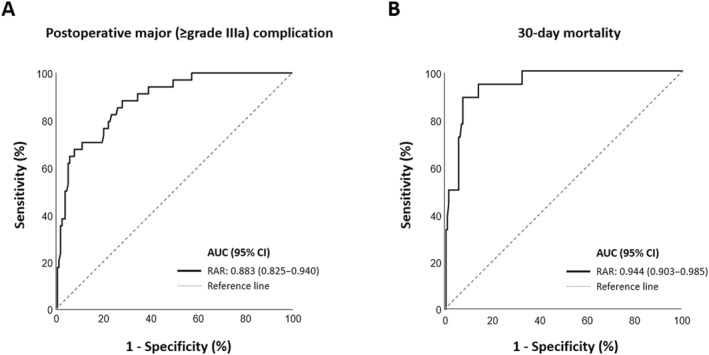

## 1 Introduction

The improved understanding of peptic ulcer pathogenesis, specifically the role of *Helicobacter pylori* and the advent of proton pump inhibitors, has led to a decrease in both overall peptic ulcer disease incidence and the need for surgical intervention [1, 2]. Although the incidence, hospitalization, and mortality rates related to peptic ulcer disease have significantly decreased over the past 3 decades, complications and mortality continue to be major problems for both the healthcare system and the individual patients [1, 3].

Peptic ulcer perforation remains one of the most serious complications of peptic ulcer disease, persisting as a significant surgical emergency despite the advances in medical therapy [3, 4]. Unfortunately, both morbidity (20%–50%) and mortality (10%–30%) from peptic ulcer perforation remain still high [1, 4, 5, 6, 7]. Therefore, a meticulous preoperative assessment of clinical severity and identificaton of risk factors are crucial for achieving optimal patient outcomes [8].

Recent studies have highlighted several factors that significantly influence the prognosis in cases of peptic ulcer perforation. Aside from the well‐known role of older age as a risk factor for increased morbidity and mortality, preoperative conditions such as comorbid diseases, high American Society of Anesthesiologists (ASA) score, delayed diagnosis, abnormal renal function, hypoalbuminemia, and shock on admission have been demonstrated to significantly impact the clinical course and outcomes [1, 2, 3, 4, 5, 7, 9]. Despite the knowledge of these risk factors, accurately predicting prognosis often remains elusive. Therefore, numerous scoring systems and laboratory markers, such as the Boey score and the peptic ulcer perforation (PULP) score, have been investigated as potential prognostic indicators. However, limitations persist regarding sensitivity, specificity, simplicity, and practicality in clinical practice [3, 8, 10, 11, 12, 13]. Consequently, there is a growing interest in simple, cost‐effective biomarkers that can complement existing tools and enhance the assessment and management of risk.

One promising biomarker of interest is the red cell distribution width (RDW)‐to‐albumin ratio (RAR), which has been explored for its potential predictive value in various inflammatory and critical conditions [14, 15, 16]. RDW, a measure of the variation in the size of red blood cells, has been associated with adverse outcomes in several chronic and acute diseases, whereas serum albumin levels often reflect nutritional and inflammatory status [10, 14, 17]. Recent studies have demonstrated the prognostic significance of RAR in various clinical contexts. For example, elevated RAR has been found to be associated with worse outcomes in acute pancreatitis, where it predicted disease severity and mortality with high accuracy [14]. Similarly, studies in cardiovascular disease and critical care settings, such as burn injury and sepsis, have shown that RAR is a strong predictor of mortality and complications [15, 16].

Based on the established associations between elevated RDW and decreased albumin with poorer clinical outcomes, RAR may serve as a composite marker, reflecting prognosis in peptic ulcer perforation. The objective of this study was to explore the predictive value of RAR for clinical outcomes in patients with peptic ulcer perforation. Given the significant morbidity and mortality associated with peptic ulcer perforation, identifying basic and reliable prognostic markers may help facilitate accurate early risk stratification and guide clinical decision‐making.

## 2 Material and Methods

### 2.1 Study Design and Patients

Following institutional research ethics committee approval (decision number: 2022/11), a retrospective review of patients surgically treated for perforated peptic ulcer at a tertiary care center was conducted between January 2016 and August 2024. All adult patients were identified from the hospital electronic database. Patients with malignant ulcer perforation or traumatic perforation and patients with concomitant abdominal pathology leading to peritonitis or missing data were excluded from the study. Informed consent was obtained from all patients included in the study.

### 2.2 Data Collection and Definitions

Demographics, clinical characteristics, laboratory features, and surgical outcomes of patients were retrieved from hospital medical records. Details regarding surgical procedure and techniques were obtained from operation notes. Variables collected for each patient included age, sex, ASA score, comorbidities, disease‐ and operation‐related features, time between admission to surgery, length of hospital stay, postoperative complications, and mortality. Analyzed laboratory values included the following: preoperative serum albumin, blood urea nitrogen (BUN), C‐reactive protein (CRP), white blood cell, and RDW.

Postoperative complications within 30 days after surgery were graded based on the Clavien–Dindo classification system [18]. The presence of a grade IIIa or higher complication was considered a clinically relevant complication or major complication. The 30‐day reoperation was defined as an unplanned relaparotomy within 30 days of the index surgery and 30‐day mortality was defined as any death within 30 days of the index surgical procedure. Delayed diagnosis was defined as patients presenting more than 24 h after the onset of symptoms, whereas surgical delay was defined as a surgery occurring more than 12 h after admission [2, 4, 9, 10]. The RAR was calculated as a ratio of RDW (%) to albumin (g/dL).

### 2.3 Patient Follow‐Up and Outcomes

All patients were monitored throughout their hospital stay and subsequently followed for 1 month postoperatively to assess for potential complications or mortality. In addition, patients were scheduled for follow‐up endoscopic evaluation 6–8 weeks after discharge to confirm healing and exclude possible malignant etiology.

The primary study outcome of interest was 30‐day mortality. The secondary outcome included 30‐day postoperative complications.

### 2.4 Statistical Analysis

Normal distribution of continuous variables was evaluated with quantile‐quantile plots, histograms, and the Shapiro–Wilk test. Continuous variables were reported as median (interquartile range), whereas categorical variables were presented as numbers and percentages. Associations between variables were evaluated using the Student's *t*‐test or Wilcoxon Mann–Whitney *U* test (for continuous variables) and the Pearson χ^2^ test or Fisher's exact test (for categorical variables) where appropriate. Multivariate analysis (binary logistic regression) was performed to determine independent predictors of postoperative clinical outcomes. Variables that showed significance (*p* < 0.20) in the univariate analysis and those that were clinically important were included in the multivariate analysis. Although the linearity of logits was assessed via the Box–Tidwell procedure [19], potential multicollinearity was examined using variance inflation factor (VIF) and tolerance values. The Hosmer–Lemeshow test was used to check the goodness of fit for the multivariate model. Odds ratios (ORs) of statistically significant predictors were presented with 95% confidence intervals (CIs). The area under the curve (AUC) values of RAR for 30‐day postoperative complication and 30‐day mortality were calculated using receiver operating characteristic (ROC) curve analysis. Optimal “cut‐off” values were determined according to the Youden's index. Sensitivity, specificity, positive predictive value, and negative predictive value were calculated based on the cut‐off values of RAR. Statistical analyses were performed using Jamovi, version 2.3.2.0 (The Jamovi project, Sydney, Australia). *p* < 0.05 was considered statistically significant.

Due to the retrospective nature of the study, a formal sample size calculation was not performed, and the sample size was determined by the number of eligible patients who underwent surgical treatment for perforated peptic ulcers during the study period. A post hoc power analysis conducted to assess the adequacy of the sample size for detecting differences in RAR between mortality and nonmortality groups demonstrated a power of 99.9% (effect size: 1.3, *α* = 0.05), confirming sufficient statistical strength for the primary outcome analysis.

## 3 Results

### 3.1 Patient Characteristics

Between January 2016 and August 2024, a total of 187 patients who underwent a surgical procedure for peptic ulcer perforation were analyzed in this single‐institution retrospective study. Demographic, laboratory, and clinical characteristics of the patients are presented in Table 1. The median age was 49.7 (33.9−67.5) years with a range from 18 to 96 years; 78.6% of patients were male. Almost one‐third (32.1%) had an ASA score of III–IV. A total of 162 (82.6%) perforations were identified as being of gastric origin, whereas 25 (17.4%) were of duodenal origin. Major (≥ grade IIIa) complications were encountered in 18.1% (34 of 187) of the patients. The 30‐day reoperation rate was 5.3% and the 30‐day mortality rate was 9.6%.

**TABLE 1:** Demographic, clinical, and laboratory characteristics of patients with peptic ulcer perforation.

	*n* = 187
Age, years	49.7 (33.9–67.5)
Male sex, *n* (%)	147 (78.6%)
ASA score 3–5, *n* (%)	60 (32.1%)
Cardiovascular disease, *n* (%)	35 (18.7%)
Diabetes mellitus, *n* (%)	25 (13.4%)
Chronic obstructive pulmonary disease, *n* (%)	34 (18.2%)
Delayed diagnosis, *n* (%)	53 (28.3%)
Time between admission to surgery, hours	7 (5–11)
Surgical delay, *n* (%)	35 (18.7%)
Gastric ulcer perforation, *n* (%)	162 (82.6%)
White blood cell, × 10^9^/L	12 (8.8–16.7)
C‐reactive protein, mg/L	30 (7–138)
BUN, mg/dL	17.3 (14.0–27.5)
Red cell distribution width (RDW), %	13.7 (13.2–14.9)
Albumin, g/dL	3.9 (3.4–4.1)
RAR, %/(g/dL)	3.7 (3.3–4.3)
Length of hospital stay, days	7 (6–9)
Major (≥ grade IIIa) complication, *n* (%)	34 (18.1%)
30‐day reoperation, *n* (%)	10 (5.3%)
30‐day mortality, *n* (%)	18 (9.6%)

Abbreviations: ASA, American Society of Anesthesiologists; BUN, blood urea nitrogen; ICU, intensive care unit; RAR, red cell distribution width‐to‐albumin ratio.

### 3.2 Comparative Analysis of Patient Characteristics With Respect to Postoperative Complications and 30‐Day Mortality

Patients with major complications were found to be significantly older (*p* < 0.001), predominantly female (35.3% vs. 18.3%; *p* = 0.038), and more likely to be ASA 3–5 (*p* < 0.001). The patients were more likely to have cardiovascular disease (*p* < 0.001) and chronic obstructive pulmonary disease (COPD) (*p* = 0.007) and more likely to have a delayed diagnosis (*p* = 0.011) and surgical delay (*p* = 0.003). In addition, CRP (*p* < 0.001), BUN (*p* < 0.001), RDW (*p* < 0.001), and RAR (*p* < 0.001) levels were statistically significantly higher, and white blood cell (*p* < 0.001) and albumin (*p* < 0.001) levels were significantly lower in patients with major complications (Table 2).

**TABLE 2:** Comparative analysis of patient characteristics in terms of postoperative major (≥ grade IIIa) complications.

	No or minor complication (*n* = 153)	Major (≥ grade IIIa) complication (*n* = 34)	*p* value
Age, years	44.1 (30.9–60.3)	74.4 (61.5–85.0)	< 0.001
Female sex, *n* (%)	28 (18.3%)	12 (35.3%)	0.038
ASA score 3–5, *n* (%)	31 (20.3%)	29 (85.3%)	< 0.001
Cardiovascular disease, *n* (%)	22 (14.4%)	13 (38.2%)	0.003
Diabetes mellitus, *n* (%)	19 (12.5%)	6 (17.6%)	0.426
Chronic obstructive pulmonary disease, *n* (%)	22 (14.4%)	12 (35.3%)	0.007
Delayed diagnosis, *n* (%)	37 (24.2%)	16 (47.1%)	0.011
Time between admission to surgery, hours	6.6 (4.8–10.4)	8.0 (6.4–15.3)	0.016
Surgical delay, *n* (%)	22 (14.4%)	13 (38.2%)	0.003
Gastric ulcer perforation, *n* (%)	134 (87.6%)	28 (82.4%)	0.418
White blood cell, × 10^9^/L	12.8 (9.3–17.2)	8.9 (5.7–11.8)	< 0.001
C‐reactive protein, mg/L	19.5 (5.4–99.0)	119.8 (75.5–220.9)	< 0.001
BUN, mg/dL	16.8 (13.3–23.1)	34.5 (18.6–53.1)	< 0.001
Red cell distribution width (RDW), %	13.6 (13.2–14.5)	15.0 (13.8–18.0)	< 0.001
Albumin, g/dL	4.0 (3.6–4.2)	2.9 (2.4–3.4)	< 0.001
RAR, %/(g/dL)	3.5 (3.2–4.0)	5.5 (4.2–6.9)	< 0.001
Length of hospital stay, days	7 (6–8)	14.5 (5–29)	0.001
30‐day reoperation, *n* (%)	0 (0%)	10 (29.4%)	< 0.001
30‐day mortality, *n* (%)	0 (0%)	18 (52.9%)	< 0.001

Abbreviations: ASA, American Society of Anesthesiologists; BUN, blood urea nitrogen; RAR, red cell distribution width‐to‐albumin ratio.

A comparative analysis of patients in terms of 30‐day mortality following surgical procedures for peptic ulcer perforation is presented in Table 3.

**TABLE 3:** Comparative analysis of patient characteristics in terms of 30‐day mortality after a surgical procedure for peptic ulcer perforation.

	Alive at 30 days (*n* = 169)	30‐day mortality (*n* = 18)	*p* value
Age, years	47.9 (32.6–61.8)	83.3 (74.7–87.3)	< 0.001
Female sex, *n* (%)	33 (19.5%)	7 (38.9%)	0.057
ASA score 3–5, *n* (%)	43 (25.4%)	17 (94.4%)	< 0.001
Cardiovascular disease, *n* (%)	24 (14.2%)	11 (61.1%)	< 0.001
Diabetes mellitus, *n* (%)	21 (12.5%)	4 (22.2%)	0.250
Chronic obstructive pulmonary disease, *n* (%)	26 (15.4%)	8 (44.4%)	0.002
Delayed diagnosis, *n* (%)	44 (26.0%)	9 (50.0%)	0.051
Time between admission to surgery, hours	7.0 (5.0–10.5)	8.0 (4.1–15.3)	0.356
Surgical delay, *n* (%)	29 (17.2%)	6 (33.3%)	0.094
Gastric ulcer perforation, *n* (%)	148 (87.6%)	14 (77.8%)	0.246
White blood cell, × 10^9^/L	12.7 (9.1–16.9)	8.6 (4.6–11.4)	< 0.001
C‐reactive protein, mg/L	24.5 (6.1–128.1)	117.2 (74.9–190.0)	0.001
BUN, mg/dL	16.8 (13.5–24.3)	39.4 (29.9–55.3)	< 0.001
Red cell distribution width (RDW), %	13.7 (13.2–14.5)	17.3 (14.7–18.7)	< 0.001
Albumin, g/dL	3.9 (3.5–4.2)	2.7 (2.2–3.2)	< 0.001
RAR, %/(g/dL)	3.5 (3.2–4.1)	6.4 (5.4–8.5)	< 0.001
Length of hospital stay, days	7 (6–9)	6 (2–16)	0.155
Major (≥ grade IIIa) complication, *n* (%)	16 (9.5%)	18 (100%)	< 0.001
30‐day reoperation, *n* (%)	5 (3.0%)	5 (27.8%)	< 0.001

Abbreviations: ASA, American Society of Anesthesiologists; BUN, blood urea nitrogen; RAR, red cell distribution width‐to‐albumin ratio.

### 3.3 Independent Factors of Postoperative Complications and 30‐Day Mortality

Univariate regression analysis revealed that age, sex, cardiovascular disease, COPD, delayed diagnosis, surgical delay, white blood cell, CRP, BUN, and RAR were associated with a significantly higher risk of developing major complications. On multivariate analysis, factors that remained significantly associated with postoperative major complications included age (OR 1.08, 95% CI 1.04−1.13, *p* < 0.001), surgical delay (OR 4.78, 95% CI 1.24−18.51, *p* = 0.023), CRP (OR 1.01, 95% CI 1.00−1.02, *p* = 0.036), and RAR (OR 2.53, 95% CI 1.58−4.07, *p* < 0.001) (Table 4).

**TABLE 4:** Multivariate analysis for predictors of postoperative major (≥ grade IIIa) complications after peptic ulcer perforation.

	Univariate analysis		Multivariate analysis	
	OR (95% CI)	*p* value	OR (95% CI)	*p* value
Age, years	1.08 (1.05–1.11)	< 0.001	1.08 (1.04–1.13)	< 0.001
Female sex, *n* (%)	2.44 (1.08–5.49)	0.032	—	—
Cardiovascular disease, *n* (%)	3.69 (1.61–8.42)	0.002	—	—
COPD, *n* (%)	3.25 (1.41–7.49)	0.006	—	—
Delayed diagnosis, *n* (%)	2.79 (1.29–6.01)	0.009	—	—
Surgical delay, *n* (%)	3.69 (1.61–8.42)	0.002	4.78 (1.24–18.51)	0.023
White blood cell, × 10^9^/L	0.88 (0.82–0.96)	0.003	—	—
C‐reactive protein, mg/L	1.01 (1.00–1.01)	< 0.001	1.01 (1.00–1.02)	0.036
BUN, mg/dL	1.07 (1.05–1.10)	< 0.001	—	—
RAR, %/(g/dL)	2.81 (1.95–4.03)	< 0.001	2.53 (1.58–4.07)	< 0.001

Abbreviations: ASA, American Society of Anesthesiologists; BUN, blood urea nitrogen; CI, confidence interval; COPD, chronic obstructive pulmonary disease; OR, odds ratio; RAR, red cell distribution width‐to‐albumin ratio.

Factors that were associated with 30‐day mortality on univariate analysis included age, sex, cardiovascular disease, COPD, delayed diagnosis, surgical delay, white blood cell, CRP, BUN, and RAR. On multivariate analysis, only age (OR 1.17, 95% CI 1.06−1.29, *p* = 0.003) and RAR (OR 2.94, 95% CI 1.56−5.54, *p* = 0.001) remained independent predictors of mortality (Table 5).

**TABLE 5:** Multivariate analysis for predictors of 30‐day mortality after surgery for peptic ulcer perforation.

	Univariate analysis		Multivariate analysis	
	OR (95% CI)	*p* value	OR (95% CI)	*p* value
Age, years	1.13 (1.07–1.18)	< 0.001	1.17 (1.06–1.29)	0.003
Female sex, *n* (%)	2.62 (0.95–7.28)	0.064	—	—
Cardiovascular disease, *n* (%)	9.49 (3.35–26.90)	< 0.001	—	—
COPD, *n* (%)	4.40 (1.59–12.19)	0.004	—	—
Delayed diagnosis, *n* (%)	2.84 (1.06–7.61)	0.038	—	—
Surgical delay, *n* (%)	2.41 (0.84–6.96)	0.103	—	—
White blood cell, × 10^9^/L	0.83 (0.74–0.94)	0.002	—	—
C‐reactive protein, mg/L	1.01 (1.00–1.01)	0.008	—	—
BUN, mg/dL	1.08 (1.05–1.11)	< 0.001	—	—
RAR, %/(g/dL)	2.99 (2.00–4.46)	< 0.001	2.94 (1.56–5.54)	0.001

Abbreviations: ASA, American Society of Anesthesiologists; BUN, blood urea nitrogen; CI, confidence interval; COPD, chronic obstructive pulmonary disease; OR, odds ratio; RAR, red cell distribution width‐to‐albumin ratio.

### 3.4 Diagnostic Accuracy of RAR for Predicting Postoperative Complications and 30‐Day Mortality

**FIGURE 1:**
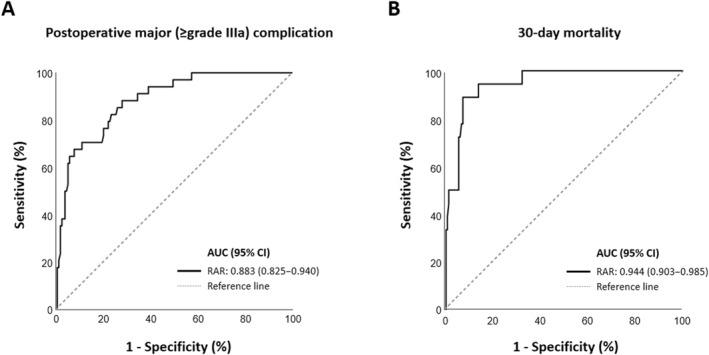
Predictive performance of RAR for major complications and 30‐day mortality by receiver operating characteristic curves.

Table 6 and Figure 1 show predictive performance of RAR for major complications and 30‐day mortality. The optimal cut‐off value of RAR for the evaluation of complications was calculated to be 3.9 (AUC = 0.883), with a sensitivity of 88.2% and a specificity of 71.9%. For 30‐day mortality, at the optimal cut off value of 5.2 (AUC = 0.944), the sensitivity was 88.9% and the specificity was 92.3%.

**TABLE 6:** Predictive performances of RAR for assessing postoperative major (≥ grade IIIa) complications and 30‐day mortality.

	Cut‐off value	AUC (95% CI)	Sensitivity, %	Specificity, %	PPV, %	NPV, %	PLR	NLR
Postoperative major complications								
RAR, %/(g/dL)	3.9	0.883 (0.825–0.940)	88.2	71.9	41.1	96.5	3.14	0.16
30‐day mortality								
RAR, %/(g/dL)	5.2	0.944 (0.903–0.985)	88.9	92.3	55.2	98.7	11.56	0.12

Abbreviations: AUC, area under curve; CI, confidence interval; NLR, negative likelihood ratio; NPV, negative predictive value; PLR, positive likelihood ratio; PPV, positive predictive value; RAR, red cell distribution width‐to‐albumin ratio.

## 4 Discussion

This retrospective study investigated the prognostic value of the red cell distribution width (RDW)‐to‐albumin ratio (RAR) in patients with peptic ulcer perforation. Our findings demonstrated that RAR was a significant independent predictor of both major postoperative complications and 30‐day mortality in this patient cohort. Additional predictors of 30‐day mortality included increasing age, whereas additional factors contributing to postoperative complications were increased age, surgical delay, and high CRP levels.

Identifying patients at high risk for postoperative complications, morbidity, and mortality in peptic ulcer perforation remains challenging, as no single reliable factor predicts a poor outcome [3, 8, 10, 13, 20]. Despite the development of several scoring systems, including Boey, PULP, qSOFA, APACHE II, MPM II, and ASA scores, none have been widely adopted in clinical practice due to issues of complexity, nonspecificity, and variations in accuracy for outcome prediction. Furthermore, most of these scoring systems require further validation [10, 13]. Notably, these systems, which are composed of numerous physiological measurements, clinical factors, or physical health parameters, were originally designed to categorize intensive care unit patients according to risk, with the exception of the Boey and PULP scores [13]. These scoring systems also lack the use of laboratory parameters, especially those that have been found in previous studies to be an independent factor, such as albumin, BUN, or CRP [3, 10, 11, 20]. Hence, finding novel and basic indicators to complement these scoring systems is of crucial clinical value.

RDW measures the variability in the size of circulating erythrocytes, which often increases due to inflammation or impaired erythropoiesis, and is associated with an elevated risk of mortality and adverse outcomes in several conditions, including cardiovascular disease and chronic kidney disease [14, 17]. Serum albumin reflects the nutritional status and inflammatory response. Low albumin levels are independently associated with an increased risk of all‐cause and cardiovascular mortality, as well as a poor prognosis for several diseases [11, 14, 15, 16, 21]. When integrated as RAR, these two biomarkers provide a comprehensive measure of physiological dysfunction, enhancing the prognostic power of either marker alone. In a cohort study using data from the US National Health and Nutrition Examination Survey (NHANES) (*n* = 50,622) and the UK Biobank (*n* = 418,950), elevated RAR was significantly associated with increased risk of all‐cause mortality [21]. For instance, in the NHANES cohort, an elevated RAR was associated with a 1.83‐fold increase in the risk of all‐cause mortality. Additionally, RAR was found to be predictive of malignant neoplasms and other causes of mortality. These findings suggest that RAR is not only a reliable indicator of overall mortality risk but also a robust predictor of cause‐specific mortality in clinical practice​.

To date, no study has investigated the relationship between peptic ulcer perforation and RAR, but many studies have shown hypoalbuminemia to be a prognostic factor associated with a poor prognosis [3, 10, 13]. In a study by Menekse et al. [11], the POMPP scoring system was developed based on age, BUN, and albumin levels as key predictors of mortality in patients with perforated peptic ulcers. The system demonstrated a strong predictive performance, with an AUC of 0.931, a sensitivity of 82.6%, and a specificity of 89.2%, offering a potentially useful, practical, and effective tool for clinical assessment. The only study to assess the diagnostic accuracy of RDW in peptic ulcer perforation was conducted by Akturk et al. [17], and the authors found that at a cut‐off value of 14.25%, the AUC, sensitivity, and specificity of RDW as a diagnostic marker were 0.812, 80.0%, and 82.2%, respectively.

One of the key findings of this study is the strong association between RAR and outcomes after surgical treatment for peptic ulcer perforation. On multivariate analysis, RAR was found to be independently associated with a more than twofold increase in major complications and a nearly threefold increase in 30‐day mortality. The predictive performance of RAR was also notable, with an optimal cut off of 3.9 showing high sensitivity (88.2%) and specificity (71.9%) for predicting major complications. Similarly, a cut off of 5.2 predicted 30‐day mortality with a high sensitivity (88.9%) and specificity (92.3%). These findings suggest that RAR, a simple and readily available inflammation‐ and nutrition‐based marker, has a significant prognostic value and may play a role in risk stratification and clinical decision‐making. Moreover, delayed surgery and elevated CRP levels were also significant independent predictors of major complications, further emphasizing the role of systemic inflammation and timely surgical intervention in patient outcomes. However, in the context of mortality, only age and RAR remained significant in the multivariate model, suggesting that while inflammatory and surgical delay factors play a role in complications, increased age and overall physiological deterioration (as reflected by RAR) are more critical determinants of mortality risk.

Despite the promising results, there are several limitations to this study that must be acknowledged. First, this was a retrospective, single‐center study, which may limit the generalizability of the findings. Additionally, although RAR showed a strong predictive power, its utility as a prognostic marker in clinical practice requires further validation in larger, prospective studies. The lack of direct comparisons with other established scoring systems is another limitation, and future studies should assess how RAR performs relative to these tools in predicting outcomes in patients with peptic ulcer perforation. Finally, while RAR was a significant predictor of major complications and mortality, the underlying mechanisms linking elevated RDW and hypoalbuminemia to poor outcomes remain unclear. Further research into the pathophysiological basis of these associations is warranted to fully elucidate the role of RAR in the clinical course of peptic ulcer perforation.

## 5 Conclusion

Overall, this study highlights the prognostic importance of RAR in patients undergoing surgery for peptic ulcer perforation. Its strong association with both major complications and mortality, combined with its high diagnostic accuracy, suggests that RAR could be a valuable tool for clinicians in the preoperative assessment and postoperative management of these patients. Future research should focus on validating these findings in larger, multicenter cohorts and exploring the integration of RAR into standardized risk assessment protocols. By facilitating early identification of high‐risk patients, RAR could contribute to improved outcomes in this challenging clinical setting.

## Author Contributions

**Suleyman Utku Celik:** conceptualization, formal analysis, funding acquisition, investigation, methodology, project administration, resources, software, supervision, visualization, writing–original draft, writing–review and editing. **Yasin Gulap:** data curation, investigation, writing–original draft, writing–review and editing. **Mehmet Bahadir Demir:** data curation, investigation, writing–original draft, writing–review and editing. **Mehmet Mert Demircioglu:** data curation, investigation, writing–original draft, writing–review and editing. **Hilmi Erencan Polat:** data curation, investigation, writing–original draft, writing–review and editing. **Sacit Altug Kesikli:** formal analysis, investigation, methodology, resources, supervision, writing–original draft, writing–review and editing.

## Ethics Statement

The study was reviewed and approved by the institutional research ethics committee approval from Gulhane Training and Research Hospital.

## Consent

Informed consent was obtained from all patients included in this study.

## Conflicts of Interest

The authors declare no conflicts of interest.

## Conference Information

This paper is based on work previously presented at the 23rd National Surgery Congress in April 24–28 2024, Antalya, Turkey.

## References

[1] A. Shreya , S. Sahla , B. Gurushankari , et al. “Spectrum of Perforated Peptic Ulcer Disease in a Tertiary Care Hospital in South India: Predictors of Morbidity and Mortality,” ANZ Journal of Surgery 94, no. 3 (2024): 366–370, 10.1111/ans.18831. 38115644

[2] J. Y. Lau , J. Sung , C. Hill , C. Henderson , C. W. Howden , and D. C. Metz , “Systematic Review of the Epidemiology of Complicated Peptic Ulcer Disease: Incidence, Recurrence, Risk Factors and Mortality,” Digestion 84, no. 2 (2011): 102–113, 10.1159/000323958. 21494041

[3] A. Tarasconi , F. Coccolini , W. L. Biffl , et al. “Perforated and Bleeding Peptic Ulcer: WSES Guidelines,” World Journal of Emergency Surgery 15, no. 1 (2020): 3, 10.1186/s13017-019-0283-9. 31921329 PMC6947898

[4] P. Sivaram and A. Sreekumar , “Preoperative Factors Influencing Mortality and Morbidity in Peptic Ulcer Perforation,” European Journal of Trauma and Emergency Surgery 44, no. 2 (2018): 251–257, 10.1007/s00068-017-0777-7. 28258286

[5] B. Kocer , S. Surmeli , C. Solak , et al. “Factors Affecting Mortality and Morbidity in Patients With Peptic Ulcer Perforation,” Journal of Gastroenterology and Hepatology 22, no. 4 (2007): 565–570, 10.1111/j.1440-1746.2006.04500.x. 17376052

[6] P. Dogra , R. Kaushik , S. Singh , and S. Bhardwaj , “Risk Factors for Leak After Omentopexy for Duodenal Ulcer Perforations,” European Journal of Trauma and Emergency Surgery 49, no. 2 (2023): 1163–1167, 10.1007/s00068-022-02058-y. 35870005

[7] M. Abouelazayem , R. Jain , M. S. J. Wilson , et al. “Global 30‐Day Morbidity and Mortality of Surgery for Perforated Peptic Ulcer: GRACE Study,” Surgical Endoscopy 38, no. 8 (2024): 4402–4414, 10.1007/s00464-024-10881-0. 38886232

[8] A. Koranne , K. G. Byakodi , V. Teggimani , et al. “Comparative Study Between Peptic Ulcer Perforation Score, Mannheim Peritonitis Index, ASA Score, and Jabalpur Score in Predicting the Mortality in Perforated Peptic Ulcers,” Surgery Journal 8, no. 3 (2022): e162–e168. 35928546 10.1055/s-0042-1743526 PMC9345676

[9] D. L. Buck , M. Vester‐Andersen , and M. H. Møller , “Surgical Delay Is a Critical Determinant of Survival in Perforated Peptic Ulcer,” British Journal of Surgery 100, no. 8 (2013): 1045–1049, 10.1002/bjs.9175. 23754645

[10] K. Thorsen , J. A. Søreide , and K. Søreide , “What Is the Best Predictor of Mortality in Perforated Peptic Ulcer Disease? A Population‐Based, Multivariable Regression Analysis Including Three Clinical Scoring Systems,” Journal of Gastrointestinal Surgery 18, no. 7 (2014): 1261–1268, 10.1007/s11605-014-2485-5. 24610235 PMC4057623

[11] E. Menekse , B. Kocer , R. Topcu , A. Olmez , M. Tez , and C. Kayaalp , “A Practical Scoring System to Predict Mortality in Patients With Perforated Peptic Ulcer,” World Journal of Emergency Surgery 10, no. 1 (2015): 7, 10.1186/s13017-015-0008-7. 25722739 PMC4341864

[12] S. Patel , D. Kalra , S. Kacheriwala , M. Shah , and D. Duttaroy , “Validation of Prognostic Scoring Systems for Predicting 30‐Day Mortality in Perforated Peptic Ulcer Disease,” Turkish Journal of Surgery 35, no. 4 (2019): 252–258, 10.5578/turkjsurg.4211. 32551420 PMC7282445

[13] K. Thorsen , J. A. Søreide , and K. Søreide , “Scoring Systems for Outcome Prediction in Patients With Perforated Peptic Ulcer,” Scandinavian Journal of Trauma, Resuscitation and Emergency Medicine 21, no. 1 (2013): 25, 10.1186/1757-7241-21-25. 23574922 PMC3626602

[14] F. Acehan , M. Aslan , M. S. Demir , et al. “The Red Cell Distribution Width‐To‐Albumin Ratio: A Simple Index Has High Predictive Accuracy for Clinical Outcomes in Patients With Acute Pancreatitis,” Pancreatology 24, no. 2 (2024): 232–240, 10.1016/j.pan.2023.12.015. 38184456

[15] Y. J. Seo , J. Yu , J.‐Y. Park , et al. “Red Cell Distribution Width/Albumin Ratio and 90‐Day Mortality After Burn Surgery,” Burns Trauma 10 (2022): tkab050, 10.1093/burnst/tkab050. 35097135 PMC8793164

[16] W. Xu , J. Huo , G. Chen , et al. “Association Between Red Blood Cell Distribution Width to Albumin Ratio and Prognosis of Patients With Sepsis: A Retrospective Cohort Study,” Frontiers in Nutrition 9 (2022): 1019502, 10.3389/fnut.2022.1019502. 36211519 PMC9539557

[17] O. M. Akturk , M. Çakır , Y. M. Vardar , D. Yıldırım , and M. Akıncı , “A Fast and Reliable Method to Interpret Short‐Term Mortality in Perforated Peptic Ulcer: Red Cell Distribution Width Is Sensitive and Specific,” Surgery Research and Practice 2021 (2021): 5542619, 10.1155/2021/5542619. 34056058 PMC8149252

[18] D. Dindo , N. Demartines , and P.‐A. Clavien , “Classification of Surgical Complications: A New Proposal With Evaluation in a Cohort of 6336 Patients and Results of a Survey,” Annals of Surgery 240, no. 2 (2004): 205–213, 10.1097/01.sla.0000133083.54934.ae. 15273542 PMC1360123

[19] G. E. P. Box and P. W. Tidwell , “Transformation of the Independent Variables,” Technometrics 4, no. 4 (1962): 531–550, 10.2307/1266288.

[20] K. Søreide , K. Thorsen , E. M. Harrison , et al. “Perforated Peptic Ulcer,” Lancet 386, no. 10000 (2015): 1288–1298, 10.1016/s0140-6736(15)00276-7. 26460663 PMC4618390

[21] M. Hao , S. Jiang , J. Tang , et al. “Ratio of Red Blood Cell Distribution Width to Albumin Level and Risk of Mortality,” JAMA Network Open 7, no. 5 (2024): e2413213, 10.1001/jamanetworkopen.2024.13213. 38805227 PMC11134218

